# Elimination of HCV as a public health concern among people who inject drugs by 2030 – What will it take to get there?

**DOI:** 10.7448/IAS.20.1.22146

**Published:** 2017-07-28

**Authors:** Jason Grebely, Gregory J. Dore, Sébastien Morin, Jürgen K. Rockstroh, Marina B. Klein

**Affiliations:** ^a^ The Kirby Institute, UNSW Sydney, Sydney, Australia; ^b^ Executive Board, International Network on Hepatitis in Substance Users, Zurich, Switzerland; ^c^ HIV Programmes and Advocacy, International AIDS Society, Geneva, Switzerland; ^d^ Department of Medicine I, University Hospital Bonn, Bonn, Germany; ^e^ Governing Council, International AIDS Society, Geneva, Switzerland; ^f^ Chronic Viral Illness Service, McGill University Health Centre, Montreal, Canada

**Keywords:** HIV, HCV, elimination, drug users, hepatitis C, control, NSP, OST

## Abstract

**Introduction**: Globally, there is a considerable burden of HCV and HIV infections among people who inject drugs (PWID) and transmission of both infections continues. Needle and syringe programme (NSP) and opioid substitution therapy (OST) coverage remains low, despite evidence demonstrating their prevention benefit. Direct-acting antiviral therapies (DAA) with HCV cure >95% among PWID provide an opportunity to reverse rising trends in HCV-related morbidity and mortality and reduce incidence. However, HCV testing, linkage to care, and treatment remain low due to health system, provider, societal, and patient barriers. Between 2015 and 2030, WHO targets include reducing new HCV infections by 80% and HCV deaths by 65%, and increasing HCV diagnoses from <5% to 90% and number of eligible persons receiving HCV treatment from <1% to 80%. This commentary discusses why PWID should be considered as a priority population in these efforts, reasons why this goal could be attainable among PWID, challenges that need to be overcome, and key recommendations for action.

**Discussion**: Challenges to HCV elimination as a global health concern among PWID include poor global coverage of harm reduction services, restrictive drug policies and criminalization of drug use, poor access to health services, low HCV testing, linkage to care and treatment, restrictions for accessing DAA therapy, and the lack of national strategies and government investment to support WHO elimination goals. Key recommendations for action include reforming drug policies (decriminalization of drug use and/or possession, or providing alternatives to imprisonment for PWID; decriminalization of the use and provision of sterile needles-syringes; and legalization of OST for people who are opioid dependent), scaling up and improving funding for harm reduction services, making health services accessible for PWID, supporting community empowerment and community-based programmes, improving access to affordable diagnostics and medicines, and eliminating stigma, discrimination, and violence against PWID.

**Conclusions**: The ambitious targets for HCV elimination set by WHO are achievable in many countries, but will require researchers, healthcare providers, policy makers, affected communities, advocates, the pharmaceutical and diagnostics industries, and governments around the world to work together to make this happen.

## Introduction

Globally, morbidity and mortality due to hepatitis C virus (HCV) infection continues to rise [1]. People who inject drugs (PWID) represent a priority population, given the high prevalence and incidence of HCV infection observed among PWID [2–6]. Given similar modes of acquisition through sharing of needles/syringes and other injecting equipment, HIV infection is a major co-morbidity among PWID with HCV infection [7].

People who inject drugs include those who have injected an illicit drug at least once in their life. This population consists of both former injectors having ceased injecting and “recent” injectors (with definitions for “recent” varying in the literature from one month to one year) [8]. Among people with a history of injecting, a population of people receiving opioid substitution therapy (OST) for opioid dependence also exists, some of whom may continue to inject drugs [8].

In the interferon-era, diagnosis and treatment for HCV infection remained low [9–12], including among PWID [13,14], due to patient, provider, health system, and societal barriers. The availability of simple and tolerable direct-acting antiviral (DAA) therapies for HCV infection with cure rates >95% is one of the greatest medical advances in decades [15,16]. This has brought considerable optimism to people working in HCV. WHO has set an ambitious goal to eliminate HCV as a major public health threat by 2030 [17]. Between 2015 and 2030, the WHO targets include reducing new HCV infections by 80%, and the number of HCV deaths by 65%, and increasing HCV diagnoses from <5% to 90% and the number of eligible persons receiving HCV treatment from <1% to 80% [17].

This commentary discusses why PWID should be considered as a priority population in these efforts, reasons why this goal could be attainable among PWID, challenges that need to be overcome, and key recommendations for action.

## Discussion

### Why should PWID be considered as a priority population in efforts to eliminate HCV as a global public health threat?

Globally, it is estimated that 71.1 million people had chronic HCV infection in 2015 [18], with 2.3 million HIV/HCV co-infected [7,19]. There is a large burden of chronic HCV infection among recent PWID (50% prevalence of chronic infection), representing an estimated 5.6 million PWID with chronic HCV infection (8% of all infections globally) [2,19] and 1.4 million with HIV/HCV [7]. There is also a large, but unquantified, number of chronic HCV infections among PWID who have ceased injecting [2,20]. The majority of existing cases of HCV infection among recent PWID are concentrated in East and Southeast Asia (26%) and Eastern Europe (23%), with large populations also existing in Canada and the United States (17%), and Latin America (14%) [2].

In 2015, there were 1.7 million new HCV infections globally, with 23% attributable to current injecting drug use [19], related to the high HCV incidence among PWID in many settings [3–6], particularly in the initial years of injecting [5,21].

Global morbidity and mortality due to HCV infection is increasing, with 704,000 deaths attributed to HCV in 2013 [20]. Although drug-related mortality is significant among PWID, liver disease-related mortality is increasing due to ageing populations [22–24]. HIV also increases liver disease progression [25,26] and liver-related mortality [27–29] among PWID with HCV infection.

The Global Burden of Disease project estimated the total burden of HCV due to injecting drug use in 2013 as measured by disability adjusted life years (including recent and former PWID) [30]. This modelling estimated that 39% (95% uncertainty interval, 31–43%) of HCV burden was due to HCV acquired via injecting drug use [30]. Regions in Asia jointly accounted for half of the global HCV burden attributable to injecting drug use (50%), while North America attributed 11% and eastern Europe accounted for 9% [30].

Given that a large proportion of the global HCV burden and new cases of infection occur among PWID, HCV treatment should be a priority in this population to have an impact on the HCV epidemic globally. Failure to intervene at early stages may result in more costly long-term care following the development of cirrhosis, decompensated liver disease, and liver cancer [31–33].

In addition to population-level reasons why PWID should be considered as a priority in efforts to eliminate HCV as a global public health threat, it is critical to also consider the individual-level benefits that successful HCV treatment provides, including improvements in health-related quality of life [34], decreased risk of liver disease progression [35], improved survival in people with advanced liver disease [36], and the potential to enhance engagement in drug user health.

### Why is it feasible to eliminate HCV as a major public health threat among PWID?

Pan-genotypic DAA therapies with cure rates >95% provide important tools to eliminate HCV in many settings, including among people with HCV/HIV co-infection [15,16]. Importantly, results from clinical trials have been replicated in real world studies [37].

DAA therapy has improved the feasibility of HCV treatment among PWID compared to interferon-based therapies, given DAA therapies have limited psychiatric side effects, are simpler (oral, once-daily vs. weekly injections), and shorter in duration (8–12 weeks vs. 24–48 weeks). DAA therapy is effective among PWID receiving OST [38–47], people with a history of injecting drug use [48–54], and recent PWID [55–57], including those with HCV/HIV co-infection [42–45,49,52,54]. HCV reinfection incidence among PWID is low (0.0 to 5.3/100 person-years) [58–65], with higher rates among those with ongoing injecting (4.9–6.4/100 person-year) [59,61,62,64]. Strategies to enhance HCV prevention, such as access to high-coverage needle and syringe programmes (NSP) and OST are crucial to minimize HCV reinfection risk.

In many settings, HCV transmission among PWID may occur as a result of ongoing risk behaviours, such as needle-syringe and equipment sharing [3–6]. Mathematical modelling suggests that HCV treatment as prevention (modest scale-up of DAA HCV treatment to 8 per 100 PWID) could lead to substantial reductions in HCV prevalence among PWID, thereby lowering HCV incidence and preventing transmission [66–73]. Given the potential prevention benefits, HCV treatment among PWID has enhanced cost-effectiveness [31–33]. However, scale-up of harm reduction services, such as NSP and OST, are required to maximize the benefits of DAA HCV therapy for prevention [68]. Global elimination of HCV as a public health threat among PWID will require increased coverage of NSP and OST interventions internationally.

### What challenges need to be overcome to achieve HCV elimination as a major public health threat among PWID?

#### Poor global coverage of harm reduction services

**Figure 1.**
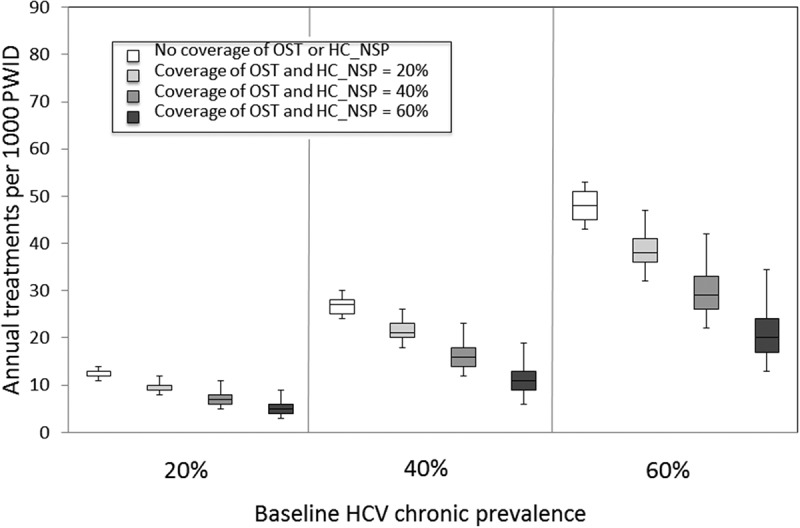
Combinations of annual treatment rates per 1000 injectors and coverage of opioid substitution therapy (OST) and high-coverage NSP (HCNSP) required to reduce prevalence by 50% within 10 years. Results shown for 3 baseline chronic prevalence settings (20%, 40%, and 60%). Assumes no intervention coverage at baseline with OST and HCNSP scale-up to 0%, 20%, 40%, or 60% of each and using direct-acting antivirals (90% SVR). The box-plots signify the uncertainty (middle line is the median, limits of the boxes are 25% and 75% percentiles and whiskers are 2.5% and 97.5% percentiles) in the impact projections due to uncertainty in the intervention effect estimates. Reproduced with permission from [68].

OST with methadone or buprenorphine is effective for the management of opioid dependence [74,75] and prevents HCV and HIV infections [76–81]. Combination OST and high-coverage NSP (adequate needles/syringes to cover all injecting episodes) can reduce HCV incidence by up to 80% [81–86]. NSP also prevents HIV infection [87]. NSP are recognized as one of the most cost-effective public health interventions [88]. Mathematical modelling studies suggest that although HCV treatment for PWID can lead to substantial reductions in HCV prevalence and reduce transmission [67,68,70–72], prevention benefits should be greatest when delivered in combination with OST and NSP (Figure 1) [68].

Globally, OST and NSP coverage are low. In 2016, among countries and territories where injecting drug use has been reported, only 57% have NSP and 51% have implemented OST [89]. Regional/national coverage varies substantially, with the highest rates of needle-syringe distribution (202 needle-syringes per PWID per year) in Australasia and the lowest rates in Latin America and the Caribbean, Middle East, and Africa (0.1–0.5 needle–syringes per PWID per year). The recommended target by WHO is 200 needles-syringes per PWID per year [90]. Western Europe has the highest levels of OST coverage (61 per 100 PWID), but low levels are observed in central Asia, Latin America and sub-Saharan Africa (1 per 100 PWID).

Despite that methadone and buprenorphine are on the WHO list of essential medicines, OST coverage is also hampered in some countries (e.g. the United States), by alternative therapies with limited evidence (e.g. naltrexone). Also, there are little data on potential substitution therapies for people who inject stimulants. Improved access to evidence-based harm reduction services will be required to support efforts to eliminate HCV infection as a global public health threat.

#### Restrictive drug policies and criminalization of drug use

The criminalization of drug use and restrictive drug policies has many unintended harms with important implications for HCV/HIV prevention and management among PWID [91–95]. In countries with restrictive drug law enforcement, the criminalization of drug use and the fear of arrest may drive people away from prevention and care services resulting in decreased needle-syringe distribution and increased needle-syringe sharing [91,94]. This represents a missed opportunity for engagement in HCV and HIV testing and treatment.

In countries with repressive drug policies, PWID often end up in prison. Among people with a history of injecting drug use in prison, the prevalence of chronic HCV infection is 48% (95% CI: 44%, 53%) [96] and incidence is 6.3–16.4 per 100-person years [97,98].

The majority of prisons lack OST, NSP, and HCV treatment services [96]. As highlighted by the Vienna Declaration [93] and Commissions in The Lancet [94,95,99], drug policy reforms such as the decriminalization of drug use and/or possession, providing alternatives to imprisonment for PWID, scale-up of evidence-based drug-dependence treatment, abolishment of compulsory drug-treatment centres, and examining the potential of offering OST, NSP and HCV therapy in prisons can improve access to HCV/HIV prevention and care for PWID which will be critical in reducing the global HCV burden.

#### Poor access to health services for PWID

People who inject drugs bear considerable stigma and discrimination, are more likely to have socio-economic disadvantage, have considerable medical morbidities, and experience disparities in access to health care. Unintended harms associated with injecting drug use include blood borne viral infections, overdose, and injecting-related bacterial infections [2,100,101]. Mortality among opioid dependent people is 14 times higher than that among people of the same age and sex [102], driven by drug-related harm and HCV-related liver disease [30]. Given that PWID face multiple medical co-morbidities, HCV infection may not always be at the top of the list of competing health priorities.

Many PWID do not access health services. In some settings, there is lack of coverage of services or barriers experienced when trying to access services (e.g., limited hours of service, long wait times, and shortage of health care practitioners) [103]. Access to good quality healthcare should be a basic human right for any person, irrespective of whether they use drugs. In other settings, a lack of engagement in health services can stem from stigma and discrimination experienced from past encounters with the health system [103–110]. In one study from Thailand, 26% of PWID reported avoiding health services, with factors associated with avoidance of healthcare including experience of verbal abuse, having been refused medical care, and having experienced previous barriers to accessing health care [105]. Even in settings where healthcare is provided without stigma or discrimination, PWID may not engage with providers out of the fear of letting down their providers (e.g., missing appointments, forgetting to get blood tests, etc.). Stigma and discrimination must be addressed in order to improve the overall health of PWID. There is good evidence to support that involving community-based PWID organizations in the design and implementation of programmes can reduce stigma and discrimination, enhance HCV/HIV prevention and care, and lead to changes in health policies [111].

#### Low HCV testing, linkage to care and treatment among PWID

Globally, HCV testing/diagnosis remains inadequate, including among PWID [9–12]. Simplified models of HCV care (including integration into existing HIV and drug health services) across a range of settings facilitates improved engagement in HCV care [112]. HCV treatment and care should be delivered in a range of settings including hospital clinics, drug treatment clinics, community health centres, primary care clinics, NSP, prisons, homelessness settings, and supervised consumption sites [112]. Providing HCV care at venues where people are accessing services should facilitate greater engagement. Multidisciplinary care that offers support for drug dependency, social and psychological services in addition to medical care (such as those developed for the treatment of HIV), nurse-led care, and the use of peers represent care models that have been successful at increasing access to HCV testing and treatment among PWID [113].

Many people have received HCV antibody testing (indicates exposure to the virus), but not HCV RNA testing (indicates active infection). Given 25% spontaneously clear HCV infection [114], enhanced diagnosis of active infection is required to improve the care cascade. Enhancing diagnosis will require cheaper, quicker, and government-reimbursed point of care diagnostics that can detect active infection in a single visit.

#### Restrictions for accessing DAA therapy

To reduce the budget impact of high-price DAA therapies, governments in many countries are restricting access to DAA therapy based on fibrosis stage, recent drug use, and prescriber type [100,115,116]. This is inconsistent with international guidelines from AASLD/IDSA, EASL, INHSU, and WHO stating that HCV treatment should be made available for everyone with HCV, irrespective of disease stage, or drug use [76,117–119].

Limiting DAA access to patients with advanced liver disease is a poor public health strategy [120]. Successful treatment of HCV infection reduces progression of liver disease [35] and lowers mortality in people with advanced liver disease [36]. Treatment of those with the greatest risk of transmission, such as PWID who may younger and have mild disease, also helps to prevent onward HCV transmission [67]. Also, engagement of PWID in HCV services may provide an opportunity to engage people in health services early in their drug using career.

The presence of restrictions limiting DAA prescribing to specialists [in Europe, 94% of countries require a specialist to prescribe a DAA [121]] is a major barrier to achieving HCV elimination. There is not enough specialist capacity to meet the treatment demand, and many PWID who require treatment are not linked to care in specialist clinics in hospitals. One approach to address this issue is broadened prescribing to allow people to get treatment where they feel most comfortable. In Australia, there are no restrictions based on disease stage or drug use; and primary care providers are eligible to prescribe. In 2016, 32,400 people were treated (~14% of people with chronic HCV), with the proportion of primary care prescribers increasing from 5% to 20% [122]. In low-income (and many middle-income) countries, there are considerable additional system-level barriers, including poor access to health services, and limited access to affordable diagnostics and DAA therapies, which need to be addressed.

DAA reimbursement restrictions in high-income countries are linked to the high price of therapies. The high demand (actual or anticipated) for DAA therapies has led national governments (and payers) to restrict access to reduce the potential impact on healthcare budgets. Nonetheless, there are a number of countries – e.g. Australia, Brazil, Canada, France, Iceland, Italy, Portugal, and Spain – that have negotiated innovative financing agreements with pharmaceutical companies which have enabled some restrictions to be removed based on volume purchasing agreements. Other countries have gained access to affordable DAAs through voluntary licenses allowing the use (through local production or importation) of generic drugs. Unfortunately, many countries remain without access to affordable DAAs, given a high HCV burden and a middle-income status which may make negotiation with drug originators difficult and exclude them from voluntary licenses.

DAA restrictions have led to prioritization of people with advanced liver disease (to reduce risk of complications of HCV-related liver disease at an individual level) in many settings. In order to achieve HCV elimination goals, people who are actively injecting (to reduce the risk of HCV transmission at a population-level) clearly need to be a further priority group. As demonstrated by mathematical modelling [66], a dual focus on these two groups is required to achieve the WHO elimination targets to reduce both new infections and mortality by 2030. Such prioritization strategies should be built upon a foundation of broad DAA access, which will require negotiations to lower DAA prices (or discounts to list prices) to facilitate removal of restrictions.

#### Lack of national strategies and government investment to support WHO elimination goals

In 2013, only 37% of WHO member states reported having national viral hepatitis strategies [123]. National viral hepatitis strategies are critical to define national priorities; outline actions; enable the effective and efficient use of resources; allocate clear roles and responsibilities to stakeholders; and enable the measurement of progress [124]. Government investment must accompany national strategies to ensure their successful implementation. As highlighted by the WHO Global Health Sector Strategy on Viral Hepatitis, 2016–2021, countries can contribute to the elimination of viral hepatitis as a global public health threat if they act with enough resolve to achieve 2030 targets [17].

## Conclusions

Key recommendations for action to eliminate HCV infection as a global health threat among PWID by 2030:

1. Reforming drug policies – Countries must consider drug policy reforms. This includes the decriminalization of drug use and/or possession, or providing alternatives to imprisonment for PWID; developing policies and laws that decriminalize the use of sterile needles/syringes (thereby permitting NSP); and legalizing OST for people who are opioid-dependent, thereby, reducing incarceration;
2. Scaling up harm reduction services – Governments and funders must improve access to harm reduction services by increasing financial support of harm reduction services and protecting funding for programmes;
3. Making health services accessible for PWID – Health services must be made available, accessible and acceptable to PWID, based on the principles of medical ethics, avoidance of stigma, non-discrimination and the right to health [111];
4. Supporting community empowerment and community-based programmes – Programmes must implement interventions to enhance community empowerment, in particular for PWID [111]. Governments and funders must also improve access to peer-based and community-based programmes led by and for PWID by increasing financial support and protecting funding for such programmes;
5. Improving access to affordable diagnostics and medicines – Advocates, researchers, healthcare providers, policy makers, and the affected community must work together to negotiate better prices for diagnostics and DAAs and work towards broadened access; and
6. Eliminating stigma, discrimination, and violence – Advocates, researchers, healthcare providers and the affected community must work together to eliminate stigma, discrimination and violence against PWID.

The ambitious targets for HCV elimination set by WHO are achievable in many countries globally, but will require researchers, healthcare providers, policy makers, the affected community, advocates, the pharmaceutical and diagnostics industries, and governments around the world to work together to make this happen.

## Abbreviations

AASLD: American Association for the Study of Liver Diseases
DAA: Direct acting antiviral
EASL: European Association for the Study of the Liver
HCNSP: High-coverage needle and syringe programmes
HCV: Hepatitis C virus
HIV: Human immunodeficiency virus
IAS: International AIDS Society
INHSU: International Network on Hepatitis in Substance Users
OST: Opioid substitution
NSP: Needle and syringe programmes
PWID: People who inject drugs
RNA: Ribonucleic acid
WHO: World Health Organization
